# The Use of Circulating Tumor DNA to Monitor and Predict Response to Treatment in Colorectal Cancer

**DOI:** 10.3389/fgene.2019.01118

**Published:** 2019-11-21

**Authors:** Mifanwy Reece, Hariti Saluja, Paul Hollington, Christos S. Karapetis, Sina Vatandoust, Graeme P. Young, Erin L. Symonds

**Affiliations:** ^1^Colorectal Surgery, Division of Surgery & Perioperative Medicine, Flinders Medical Centre, Bedford Park, SA, Australia; ^2^Department of Medicine, College of Medicine and Public Health, Flinders University, Bedford Park, SA, Australia; ^3^Flinders Centre for Innovation in Cancer, College of Medicine and Public Health, Flinders University, Bedford Park, SA, Australia; ^4^Department of Medical Oncology, Flinders Medical Centre, Bedford Park, SA, Australia; ^5 ^Bowel Health Service, Flinders Medical Centre, Bedford Park, SA, Australia

**Keywords:** circulating tumor DNA (ctDNA), colorectal cancer (CRC), mutation, methylation, surgery, chemotherapy

## Abstract

**Background:** Colorectal cancer is one of the most common cancers worldwide and has a high mortality rate following disease recurrence. Treatment efficacy is maximized by providing tailored cancer treatment, ideally involving surgical resection and personalized neoadjuvant and adjuvant therapies, including chemotherapy, radiotherapy and increasingly, targeted therapy. Early detection of recurrence or disease progression results in more treatable disease and is essential to improving survival outcomes. Recent advances in the understanding of tumor genetics have resulted in the discovery of circulating tumor DNA (ctDNA). A growing body of evidence supports the use of these sensitive biomarkers in detecting residual disease and diagnosing recurrence as well as enabling targeted and tumor-specific adjuvant therapies.

**Methods:** A literature search in Pubmed was performed to identify all original articles preceding April 2019 that utilize ctDNA for the purpose of monitoring response to colorectal cancer treatment.

**Results:** Ninety-two clinical studies were included. These studies demonstrate that ctDNA is a reliable measure of tumor burden. Studies show the utility of ctDNA in assessing the adequacy of surgical tumor clearance and changes in ctDNA levels reflect response to systemic treatments. ctDNA can be used in the selection of targeted treatments. The reappearance or increase in ctDNA, as well as the emergence of new mutations, correlates with disease recurrence, progression, and resistance to therapy, with ctDNA measurement allowing more sensitive monitoring than currently used clinical tools.

**Conclusions:** ctDNA shows enormous promise as a sensitive biomarker for monitoring response to many treatment modalities and for targeting therapy. Thus, it is emerging as a new way for guiding treatment decisions—initiating, altering, and ceasing treatments, or prompting investigation into the potential for residual disease. However, many potentially useful ctDNA markers are available and more work is needed to determine which are best suited for specific purposes and for improving specific outcomes.

## Introduction

Colorectal cancer (CRC) is the third most common cancer worldwide (Ferlay et al., 2015). Overall five-year survival is 64.4% which varies significantly depending on stage at diagnosis (89.9% for stage I–II to 14.2% for stage IV) (Howlader et al., 2018). CRC is primarily treated with surgical resection (locally advanced low rectal cancer first receiving neoadjuvant chemoradiotherapy). Adjuvant therapies are indicated for later stage disease (usually stage IIB and above) as determined by pathological tumor staging (with radiological diagnosis of metastatic disease). Pathological staging currently provides the most accurate predictor of those at greatest risk of developing disease recurrence and for whom chemotherapy is designed to reduce that risk. Nonetheless, 17–40% of curatively treated CRC will recur, with high associated mortality (Duineveld et al., 2016; Zare-Bandamiri et al., 2017). Fortunately, treatments are improving for late stage CRC, and early detection of recurrence maximizes treatment options and is associated with improved survival (Pita-Fernandez et al., 2015).

CRC surveillance currently relies upon serial radiological investigations (usually computed tomography (CT), which subjects patients to significant cumulative radiation doses and a not insignificant false positive rate) (Chao and Gibbs, 2009), and measurement of blood carcinoembryonic antigen (CEA) levels. CEA is currently the only guideline recommended blood biomarker for post-surgical monitoring of CRC, with a positive result triggering an earlier-than-scheduled CT scan. Despite its recommended use, CEA is inadequately sensitive to reliably detect recurrence; there are common patient factors known to lower specificity [e.g. smoking, infections, chronic obstructive pulmonary disease, inflammatory bowel disease, liver disease (Ruibal Morell, 1992)]; the criterion value for triggering radiological assessment is not universally agreed upon; and its use for improving survival has been questioned (Nicholson et al., 2015; Shinkins et al., 2017). Nonetheless, improved survival is more likely when distant metastatic disease is identified and promptly treated before it becomes symptomatic (Nordic Gastrointestinal Tumor Adjuvant Therapy, 1992). Advances over the past decade in surgical, radiological, and therapeutic options have increased the relative proportion amenable to curative-intent surgery (up to 50% for single organ metastases) (Vigano et al., 2018). Five-year survival of CRC patients after complete resection of liver or lung metastases can exceed 40% (Kanas et al., 2012; Gonzalez and Gervaz, 2015). Thus, early detection of lesions amenable to curative surgery is a crucial strategy for mortality reduction.

In addition to the limitations with current surveillance tools, the methods available for monitoring response to systemic therapy of CRC are suboptimal. Where systemic treatments are given in the setting of radiologically visible disease [usually metastatic CRC (mCRC)], the gold standard to evaluate response to treatment is RECIST 1.1 (Schwartz et al., 2016) (Response Evaluation Criteria in Solid Tumors 1.1) which uses regularly scheduled radiological imaging to assess changes in the longest axial diameter of tumors. However, size measurements with RECIST 1.1 focus on just one area of the tumor and do not necessarily reflect the entire tumor burden (Berger et al., 2017), and CEA has limited utility for this purpose.

There are also limitations in determining patient suitability for targeted treatment. For example, treatment with the anti-epidermal growth factor receptor (EGFR) drugs cetuximab and panitumumab is ineffective in cancers that have mutations in RAS pathway genes. These mutations, whether they are present prior to treatment or if they develop with treatment, can result in resistance to therapy. Therefore mutation profiling is essential. In current clinical practice, detection of mutations relies upon testing of tumor biopsies, which is subject to sampling error. The heterogenous and ever-evolving nature of CRC sub-clones (Siravegna et al., 2018) within an individual patient also makes this method of diagnosis of genetic mutations problematic.

To address some of the limitations with clinical monitoring, attention has turned to the measurement of cell-free circulating tumor DNA (ctDNA) in serum or plasma, also known as a “liquid biopsy” in contexts where one wishes to characterize the tumor. Both somatic and epigenetic DNA alterations occurring within cancer cells are released into the bloodstream following apoptosis or necrosis, and can be detected despite the presence of cell-free circulating DNA from normal cells. Cell-free circulating DNA is more abundant than whole circulating tumor cells, and therefore within this review we have focussed only on the use of ctDNA. While the mutation profile of each individual cancer will be unique, certain mutation patterns are associated with CRC (e.g. *APC, KRAS,* and *BRAF*). Gene mutations that have been identified within a tumor biopsy or detected in an initial panel of ctDNA genes can be used to make a personalized assay for each patient which can be utilized for ongoing ctDNA measurement. The presence of a particular gene alteration can be used to identify those who are “positive” or “negative,” or the mutant allele frequency (MAF) can be used, allowing reporting of ctDNA levels. A non-invasive measure of tumor volume and response to therapy with ctDNA, as well as a technique to personalize therapy based on changes in tumor biology, will provide a much-needed clinical monitoring tool. In addition, blood-based biomarkers that are more sensitive than CEA and CT and hence lead to earlier detection of asymptomatic recurrence, that can also predict and monitor response to standard chemotherapy and newer biological agents, are likely to have diagnostic benefits that will ultimately prolong survival.

The aim of this review was to investigate the utility of ctDNA for monitoring the response to therapy, both surgical and pharmacological intervention.

## Search Criteria and Overview

**Figure 1:**
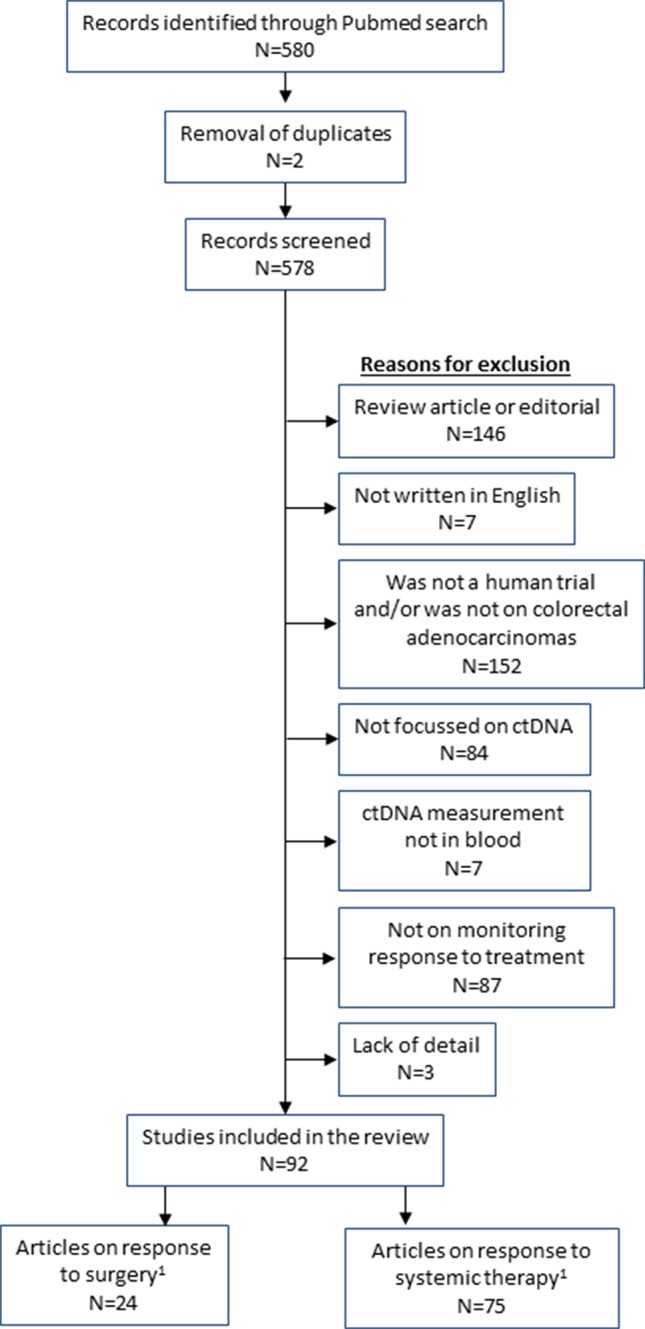
Flow diagram of studies included in the review. ^1^Total of surgery and systemic therapy studies are greater than 92 as some studies analysed both types of treatment.

The following search terms were applied in Pubmed: (“liquid biopsy” OR ctDNA OR “circulating tumor DNA” OR “tumor derived DNA” OR “circulating tumor DNA” OR “tumor derived DNA” OR “cell free DNA” OR “cell-free”) AND (“large intestine” OR colon OR caecum OR rectum OR colorect*) AND (tumor OR tumor OR malignan* OR cancer OR adenocarcinoma OR carcinoma) AND (burden OR residual OR *therapy OR load OR respon* OR treatment OR monitoring). This gave 580 results (as of 8^th^ April 2019). Papers were excluded with the following features: not an original research study (i.e. review or commentary), non-English, not a human study, not on colorectal adenocarcinoma, did not measure ctDNA, did not include ctDNA measurements in blood, did not assess ctDNA in relation to monitoring treatment (either surgery, chemotherapy or radiotherapy; prognostic studies were excluded if there was no monitoring with ctDNA). Three authors checked all papers for appropriateness for inclusion (MR, HS, and ES). This left 92 articles for review (Figure 1).

**Figure 2:**
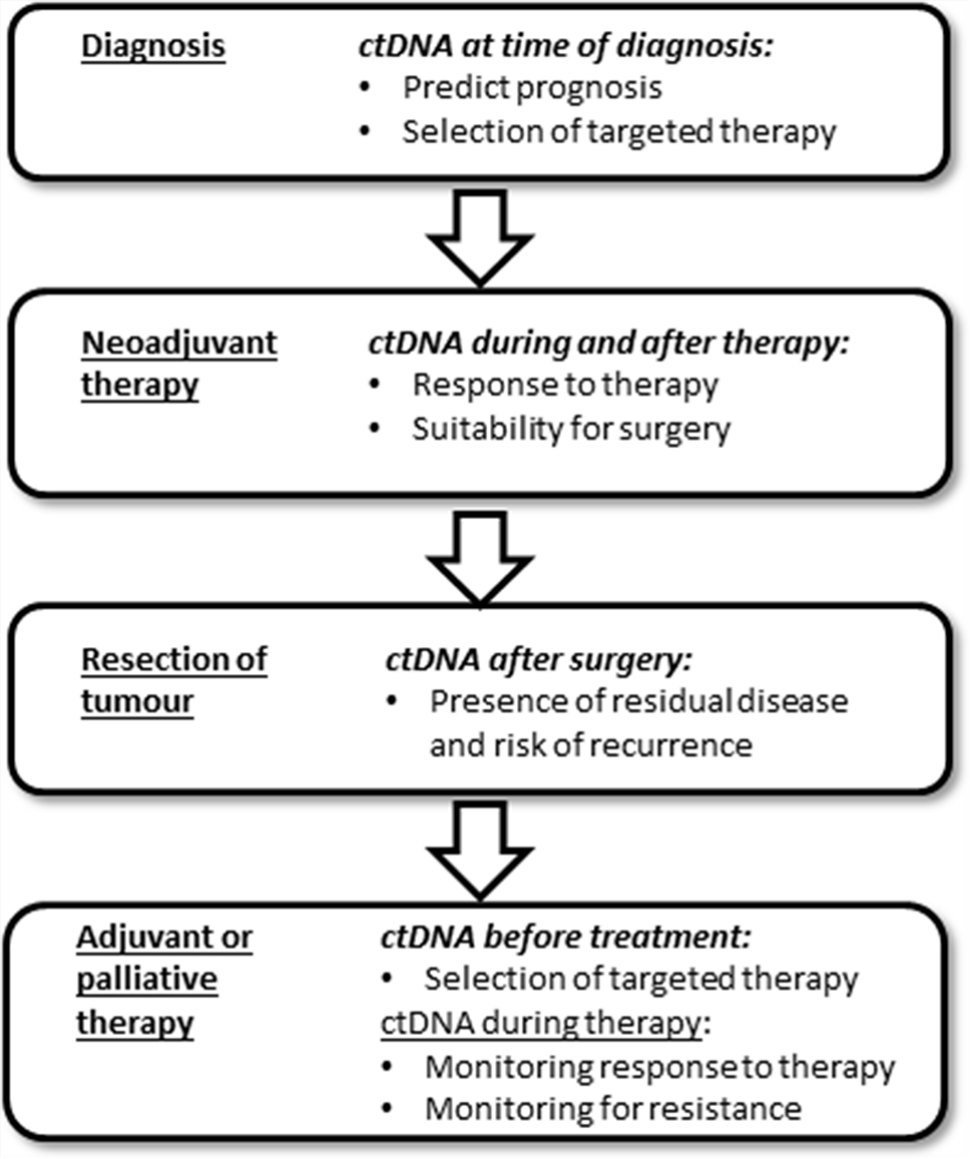
Some of the different applications of ctDNA during treatment of colorectal cancer.

This review will firstly describe the relationship of ctDNA levels to tumor burden, as well as how ctDNA has been used to assess the adequacy of surgical resection and risk of recurrence of disease. This is followed by details of the studies that have investigated the use of ctDNA in selecting therapy type, and in monitoring the response to therapy. Finally we describe how the new ctDNA tests correlate with CEA. Throughout this review the term “ctDNA” includes all DNA modifications that are present in the tumor (e.g. mutation or methylation). Figure 2 highlights some of the uses of ctDNA measurement in the patient journey, with more details provided throughout the review.

### ctDNA for Assessing Tumor Burden

An important premise for utilizing ctDNA as a tool for assessment of response to treatment is that it must be a reliable marker of tumor burden, where changes in the volume of disease (with treatment or disease progression) are reflected by changing ctDNA levels. There are a large number of studies included in this review that correlate ctDNA with tumor volume and support the use of ctDNA as a surrogate marker of tumor burden.

Many studies demonstrate the link between ctDNA and tumor burden by demonstrating a relationship between ctDNA and cancer stage. ctDNA is more likely to be detected in patients with more advanced stage cancer, whether with methylation or mutation markers. Studies have shown that ctDNA positivity increases with CRC stage, through measuring proportions of cases that are ctDNA positive with methylated *BCAT1/IKZF1* (Pedersen et al., 2015; Symonds et al., 2018), *KRAS* mutations (Shin et al., 2017), and with a panel of mutations (*KRAS, BRAF, EGFR, PIK3CA*) (Kidess et al., 2015). Measurement of the quantitative levels of ctDNA also increase with stage, as shown in a study that measured ctDNA levels of methylated *SEPT9* and *SHOX2* (Bergheim et al., 2018). The link to tumor burden is supported by studies that demonstrate significant correlations of ctDNA levels with tumor volume including r = 0.50 for ctDNA mutations (Tie et al., 2015), r = 0.74 for methylated *SEPT9* (Bhangu et al., 2018), and r = 0.75 for methylated vimentin (Overman et al., 2016); as well as studies that found that ctDNA (panel of 14 mutated genes) was strongly associated with maximum tumor diameter (p = 0.00002) and sum of tumor diameter (p = 0.00009) (Osumi et al., 2019). Patients are significantly more likely to be ctDNA positive with multiple organ metastatic disease (Osumi et al., 2019) and increasing number of lymph node metastases (Murray et al., 2018).

Studies have also shown that tumor volume changes on CT imaging mirror changes observed in ctDNA levels. In a study of 45 patients with all stages of CRC, changes in pre-operative, post-operative and surveillance ctDNA had good agreement with tumor volume on imaging and correlated with relapse (k = 0.41 p = 0.028) (Scholer et al., 2017). A positive correlation was also shown between MAF and tumor load in patients with mCRC (n = 21) receiving chemotherapy and an anti-VEGF agent (bevacizumab) (baseline r = 0.56; remission r = 0.49; post progression r = 0.75) (Yamauchi et al., 2018). In fact, the bulk of the studies included in this review, by nature of the fact that they are assessing treatment response by measuring tumor volume changes on CT and comparing these to ctDNA levels, make some mention of this correlation (either descriptively or statistically). The tables presented throughout this review highlight the papers in which tumor burden was assessed with ctDNA.

As will be outlined below, the technologies that allow for ultrasensitive detection, and the observation that ctDNA levels decrease (often to zero) following surgical resection of tumor (an intervention that immediately reduces tumor burden) adds weight to the evidence supporting ctDNA in reflecting tumor burden has enabled it to be utilized for monitoring adequacy and response to treatment.

## ctDNA for Assessing Surgical Techniques and Presence of Residual Disease

### Assessment of Adequacy of Surgical Resection

While ctDNA correlates with macroscopic tumor burden, there is emerging evidence that ctDNA may be sufficiently sensitive as to detect the presence of microscopic disease. In a study of 184 CRC patients undergoing surgery, the levels of ctDNA (using the epigenetic biomarker methylated *SEPT9*) dropped following surgery (Bergheim et al., 2018). Patients with known residual disease (metastases) were significantly more likely to remain positive post-operatively (3.1% ctDNA positive in non-metastatic disease vs 90.9% in metastatic disease, p < 0.001), as did patients with an involved resection margin (i.e. microscopic residual disease). This suggests that ctDNA may be useful as a surrogate marker for the presence of residual tumor following surgical resection, thereby assessing the adequacy of curative-intent surgery. Although TNM staging is the best current method of stratifying risk of recurrence, many patients will receive adjuvant therapy who would never develop recurrent disease, and many will not receive it because they have an early stage tumor, but will subsequently recur. Identifying patients who have microscopic residual disease should enable better estimation of risk for recurrence and enable chemotherapy to be targeted to these patients to improve survival while reducing unnecessary exposure to toxic treatments.

There have been 22 published studies that examine ctDNA levels post-operatively. Seventeen of these studies are purely descriptive (studies with n > 5 are detailed in **Supplementary Table 1**), where ctDNA fluctuations of both somatic mutations and methylation changes are described for individual or small groups of patients before and after surgery, at various time points during other neoadjuvant or adjuvant treatments, and at the time of relapse. These all report that ctDNA levels decrease (often to zero) post-operatively. As an example, Ng et al. (2017) created personalized ctDNA assays for 44 CRC patients using mutations detected in the primary tumors and used these to investigate patients peri-operatively and during subsequent treatment and follow-up. Ten out of twelve patients undergoing curative-intent surgery had ctDNA detected pre-operatively and none were positive post-operatively. One patient undergoing a palliative resection (with known residual stage IV disease) had ctDNA detected before and after surgery. The utility of ctDNA has also been shown with assessing adequacy of resection of metastatic disease. A small study measured ctDNA (mutations in *KRAS, PIK3CA, BRAF,* and *EGFR*) before and after resection of liver metastases. All four patients had a decrease in ctDNA post-surgery; three had no detectable ctDNA, but the one patient with ctDNA levels that remained high developed disease recurrence (Kidess et al., 2015).

These studies add evidence to the concept of ctDNA measuring tumor burden—once the tumor is resected, tumor burden decreases and is reflected in reduced or absent post-operative ctDNA. These descriptive studies are important proof of concept studies, which lay the foundation for the larger studies which provide more substantial evidence for the utility of ctDNA for post-surgical assessment of residual tumor burden. Many of the descriptive studies also report on examples of patients in whom post-operative ctDNA remains positive (following curative-intent surgery) and make the observation that the tumor almost always recurs and often within a short timeframe following surgery. One such study describes ctDNA changes peri-operatively in patients undergoing surgery for CRC (Diehl et al., 2008). Seventeen operations were performed with curative intent and the observed ctDNA reduction post-operatively was dramatic (median decrease of 99%). Recurrence occurred in the majority of patients (15/16) who had detectable ctDNA at first follow-up (13–56 days post operatively). None of the four patients in whom ctDNA was not detected at the first follow-up visit developed recurrence.

The remaining five studies are detailed in **Table 1** and involve a more thorough investigation of ctDNA dynamics perioperatively. They investigate CRC patients undergoing surgery for all stages of CRC. As observed in the descriptive studies, all of these studies show a decrease in ctDNA levels post-operatively. The studies that performed statistical analyses (especially relating to survival) tended to classify the detection of ctDNA as either “positive” or “negative” using a variety of ctDNA detection methods, gene mutation panels and cut-off values. Longer term follow-up analyses (usually three, but up to five years), performed in these studies investigated the impact of post-operative ctDNA positivity on prognosis.

**Table 1:** Studies assessing circulating tumor DNA (ctDNA) levels after surgery (all hazard ratios are calculated comparing those who were ctDNA positive with those who were ctDNA negative).

Reference	ctDNA type/analysis method	Sample size	Study Type	Treatment	When was blood collected	Stage (number)	Hazard ratio for recurrence	Hazard ratio for overall survival	Correlation with tumor burden
(Murray et al., 2018)	Methylated *BCAT1, IKZF1*/Real time PCR	172	Prospective	Surgery +/− chemotherapy +/− radiotherapy	Within 12 months post-surgery	I–II (93) III–IV (79)	3.8 (95% CI 1.5–9.5 p = 0.004) (multivariate)	CRC specific survival: HR 6.6 (1.9–22.8) (multivariate)	Indirectly: ctDNA positivity associated with factors that reflect tumor burden e.g. stage
(Scholer et al., 2017)	Panel of SSVs (somatic structural variants) and SPMs (somatic point mutations) including *KRAS, BRAF*/ddPCR	45	Prospective Retrospective review of CT scans	Surgery Cohort 1: longitudinal samples n = 27; Cohort 2: liver metastases treated with curative intent	Day 0 (pre-surgery), 8, 30, every 3 months until death (up to 3y)	I–III (21) IV (23) (n = 1 stage not stated)	Stages I–III (n = 21) ctDNA status post-surgery: HR 37.7 (4.2–335.5, p < 0.001) Stage IV (n = 23) ctDNA status post liver resection (curative intent): HR 4.9 (1.5–15.7, p = 0.007)	Stages I–III 5y overall survival (n = 21) ctDNA status post-surgery: HR 6.7 (1.6–28.7, P = 0.01)	Yes (n = 19) Tumor volume correlates with ctDNA levels k = 0.41 p = 0.028
(Tie et al., 2016)	Single mutation with highest MAF selected per patient/PCR with Safe-Seq	230	Prospective	Surgery Chemotherapy (n = 52)	4–10 weeks post-surgery During chemotherapy 3 monthly during follow-up n = 27	II (230)	ctDNA positivity post-surgery: HR 18 (7.9–40, p = 2.6 × 10^−12^) ctDNA positivity immediately post adjuvant chemotherapy: HR 11 (1.8–68, p = 0.001)	Not reported	Not reported
(Diehl et al., 2008)	Mutations personalized to the tumor tissue (including mutations in *APC, KRA, TP53, PIKC3A*)/PCR (BEAMing)	18	Prospective	Surgery +/− chemotherapy	Day 0, 2–10 days post-surgery, various times later	II (1) III (1) IV (16)	ctDNA positive significantly worse p = 0.006	Not reported	Indirectly: ctDNA levels decrease post operatively, & in some getting chemotherapy
(Tie et al., 2019)	1 somatic mutation selected from the most prevalent of 15 tumor tissue mutations/PCR with Safe-Seq	159	Prospective	Neoadjuvant chemoradiotherapy Surgery	Pre neoadjuvant, 4–6 weeks post neoadjuvant and 4–10 weeks post-surgery	II (35) III (124)	ctDNA positivity post neoadjuvant: HR 6.6 (2.6–17, p < 0.001) Post surgery: HR 13 (5.5–31, p < 0.001)	Not reported	Not reported

ctDNA, circulating tumor DNA; ddPCR, droplet digital PCR; HR, hazard ratio; MAF, mutant allele frequency; OS, overall survival; PFS, progression free survival; 95% CI: 95% confidence interval.

In a study of 172 post-operative CRC patients, the risk of recurrence was significantly higher for those with positive ctDNA (methylated *BCAT1/IKZF1*) within 12 months of surgery compared to those who were negative (HR 3.8, 95% CI 1.5–9.5). Close margins, apical node involvement and the presence of distant metastases were predictors of post-operative ctDNA positivity in this cohort (Murray et al., 2018). Similar findings were observed in a study of ctDNA with somatic mutations (Scholer et al., 2017). Stage I–III patients (n = 21) who had negative post-operative ctDNA had significantly higher relapse-free survival (RFS) and overall survival (OS) compared to patients who had a positive post-operative ctDNA result (RFS HR = 37.7, 95% CI 4.2–335.5; OS HR = 6.7 95% CI 1.6–28.7). This was also demonstrated in stage IV patients undergoing metastasectomy with curative intent (n = 23) who had a higher risk of relapse if their post-operative ctDNA was positive (HR = 4.9, 95% CI 1.5–15.7) (Scholer et al., 2017). High prediction of ctDNA for risk for recurrence post-surgery has also been found when ctDNA biomarkers are selected through tumor tissue analysis. In the first of these studies, 230 post-operative ctDNA levels were measured in patients with stage II colon cancer and a negative result was associated with a significantly increased RFS (HR = 18, 95% CI 7.9–40) (Tie et al., 2016). Similar outcomes were found following surgery in patients with locally advanced rectal cancer who had also received neoadjuvant chemoradiotherapy (n = 159), with a negative ctDNA result associated with low risk for relapse (HR = 13, 95% CI 5.5–31) (Tie et al., 2019).

### Methods of Surgery

Success of different methods of surgical interventions can also be assessed with ctDNA. While only two papers specifically on this topic were identified using our search criteria, evidence from studies of circulating tumor cells when comparing open versus laparoscopic surgery for CRC (Wind et al., 2009) support this idea.

Within our reviewed articles, one study investigated the use of ctDNA (mutations selected from tissue analysis) to determine if self-expanding stent placement (n = 25) or a decompression tube (n = 10) for malignant colonic obstruction treatment caused cancer cell migration (Takahashi et al., 2018). The two treatments did not differ in their clinical success, however, use of the stent was an independent predictor for increased risk for higher ctDNA concentration at day 3 compared to a decompression tube (OR 18.4, 95% CI 1.52–222). While this was a small study, the ctDNA results suggest an oncological risk associated with placement of the stent. The second study (n = 18) used measurement of ctDNA (*KRAS* and *TP53* mutations selected from tissue analysis) in the portal vein to show that tumor manipulation during surgery enhances cancer cell migration. In the conventional resection group, 73% had ctDNA detected in the portal vein, compared to only 14% in the “no-touch” isolation technique group. Monitoring of ctDNA may therefore be used to assess risk of cancer cell migration with different surgical methods.

### Summary

The studies summarized above have shown that the application of post-operative ctDNA measurement, whether with epigenetic or somatic biomarkers, shows great potential for monitoring response to surgical treatment and predicting the need for adjuvant therapy by identifying those at greatest risk of recurrence. Four of the five in-depth studies measured genetic markers and generally had higher hazard ratios for recurrence (HR range 4.9 to 37.7) than the single study examining epigenetic (methylated) markers (HR = 3.8) suggesting the potential superiority of genetic markers for this purpose (**Table 1**). These studies have also shown that patients who have ctDNA detected following (or during) surgery have an increased risk of recurrence or relapse, and have poorer survival compared to those who are negative for ctDNA. There is also the potential for using ctDNA to compare surgical methods.

While the monitoring performance and sensitivity of ctDNA for high volume disease is likely to be greater than that for microscopic disease, these studies demonstrate that the ultrasensitive detection of microscopic residual disease in the post-operative patient population can also be utilized for the detection of recurrent disease following curative treatment i.e. for longitudinal CRC surveillance. The clinical use of ctDNA for assessing for risk of microscopic residual disease has the potential to have a large impact on determining which patients may require adjuvant therapy and in the early detection of recurrence, thereby improving disease-free survival and overall survival.

## Selection of Treatment Based on ctDNA Biomarkers

The decision to provide adjuvant chemotherapy following CRC resection, and the decision on which therapeutic agent to use, may be guided by assaying for ctDNA. As described above, a post-operative positive ctDNA result increases the likelihood that residual disease is present. This result could help to determine whether adjuvant chemotherapy is needed, particularly in patients with stage II CRC where there is uncertainty regarding its benefit (Dotan and Cohen, 2011). These patients are generally not recommended adjuvant therapy, yet up to 23% will have recurrence within 5 years (Wilkinson et al., 2010). In these patients, the use of ctDNA (based on selection of a single mutation marker for each patient, personalized from tumor tissue testing) is supported by studies that showed that post-operative positive ctDNA stage II colon (Tie et al., 2016) and rectal cancer (Tie et al., 2019) patients were at very high risk for recurrence when not treated with adjuvant chemotherapy. With further research, ctDNA may provide an adjunct to the current use of TNM staging and other poor prognostic tumor factors (e.g. tumor grade, lymphovascular invasion) in determining which patients receive adjuvant therapy, with one such trial investigating the utility of ctDNA for this purpose (ANZCTR, 2017).

Measurement of certain specific ctDNA biomarkers may also be used to select appropriateness of targeted therapy. As will be described in the biological therapy section of this review, mutations in RAS pathway genes are associated with resistance to the anti-EGFR agents cetuximab and panitumumab, and therefore tumor tissue testing is performed to ensure *RAS* wild type (WT) status. However, as a result of the presence of different subclones within a tumor (or within different metastases of a patient), sampling errors may occur with solid biopsy, and therefore monitoring of ctDNA instead may reveal the complete genetic picture of tumor heterogeneity. As well as the initial determination of appropriateness of anti-EGFR for patients, through serial monitoring, ctDNA can monitor for temporal heterogeneity by detecting changes in RAS status (or other mutations) over time. A study of 11 patients with *RAS*-mutant mCRC who were progressing on anti-VEGF treatment found that four patients had converted to WT, so were commenced on anti-EGFR treatment. All four patients responded to anti-EGFR treatment with standard chemotherapy, either experiencing disease regression or stability. One of the two patients who developed significant disease progression was found to have converted back to *RAS*-mutant on ctDNA at the time of progression (Raimondi et al., 2019).

While DNA in the plasma can come from multiple tissues within the body, selecting appropriate biomarkers for ctDNA can be utilized to determine the nature of a tumor (Sun et al., 2015) and to distinguish between different types of cancer (Guo et al., 2017). This is particularly useful where there is uncertainty about the tissue of origin and this has implications for selecting appropriate treatment. Examples include using ctDNA mutations to demonstrate that ovarian tumors (Iwahashi et al., 2018) and lung metastases (Zhang et al., 2018) were of colorectal origin, enabling the appropriate selection of treatment.

## Using ctDNA to Monitor Response to Systemic Therapy

In order to provide optimal clinical management of CRC patients, it is important to regularly evaluate treatment efficacy. Ideally, this should be determined as early as possible so as to minimize exposure to ineffective treatments and limit the potential for adverse side-effects. Accessing tumor tissue directly is invasive and can be technically difficult; ctDNA might thus be an ideal way of characterising the tumor (and hence the term “liquid biopsy”). Assessment of ctDNA before, during and following therapy has the potential to determine treatment response (reduction in tumor bulk) and whether mutational status has changed which may indicate development of resistance to therapy. As previously mentioned, the current gold standard for assessing the tumor response is through imaging based on RECIST 1.1 (Schwartz et al., 2016). This, however, has poor inter-observer reproducibility, can only be applied in patients with measurable lesions, and the correlation between treatment efficacy and tumor response has been questioned (Garlan et al., 2017) as the tumor response to therapy does not necessarily predict survival in patients with mCRC (Grothey et al., 2008). It also focusses on just one region of the tumor and not the whole tumor mass. ctDNA may provide a better measure for tumor response and benefit as it has the potential to identify primary tumor biomarkers, as well as those present in metastases but not present in the primary tumor (Furuki et al., 2018). Monitoring for changes in the ctDNA profile will give opportunity for personalising treatment.

In monitoring response to therapy, the different classes of biomarker may be relevant. For instance, somatic markers are relevant to genotype and targeted therapy and are very personalized (Tie et al., 2015), while epigenetic markers are more universally relevant (Symonds et al., 2018) and while potentially useful to monitor bulk, are less applicable to targeted therapy.

A number of studies have provided evidence that ctDNA can monitor response to general systemic therapy while not focussing on a particular therapeutic agent. These studies have collectively shown that monitoring of responses to any therapy with ctDNA is reliant on the selection of appropriate ctDNA biomarkers. The majority of studies have assayed for KRAS mutations, or other mutations personalized to the tumor tissue. For example, a retrospective study that identified five CRC-specific methylated loci from cell lines showed that with longitudinal follow-up the average methylation changes reflected changes to tumor burden (Barault et al., 2018). Studies that monitored ctDNA with mutation measurements (Diehl et al., 2008; Tie et al., 2015; Berger et al., 2017; Vidal et al., 2017; Takayama et al., 2018), or with a combination of mutation and methylation ctDNA biomarkers (Boeckx et al., 2018), also showed that changes to ctDNA levels could reflect individual responses to therapies. One study demonstrated a median 99% decrease in MAF in those with a complete response, whereas those with progressive disease had a 132% increase (Vidal et al., 2017). A further study demonstrated that the presence of KRAS mutations in ctDNA was related to disease progression, particularly in the WT tumor patients treated with anti-EGFR therapy (Takayama et al., 2018). However, KRAS mutations were also detected in ctDNA in those treated with other agents such as anti-VEGF therapy, TAS-102 and regorafenib, as well as with conventional chemotherapy. A decrease in ctDNA in two patients reflected tumor shrinkage with treatment, and a stable ctDNA level suggested stable disease. However, they also observed a spike, then drop in ctDNA levels in six patients on anti-VEGF treatment or TAS-102. Rather than this increase being associated with disease progression, it was suggested that this indicated a response to the drug, as tumor morphological changes were also reported. These are similar to the findings of Tie et al. (2015), who showed that on day 3 of treatment, the spike could reflect a rapid release of DNA from the responsive tumor into the circulation.

Different ways of expressing ctDNA levels have been shown to improve clinical utility. The ability for ultrasensitive detection of ctDNA can allow sensitive changes to be monitored in a quantitative rather than qualitative fashion. One study showed that an increase in ctDNA (measured with both mutations and methylation) from pre-treatment to the start of each cycle was associated with a poorer PFS (HR 3.62), but no change to OS (Garlan et al., 2017). However, assessment of the slope of change did show an association with both PFS and OS (assessment of those with a >80% decrease in ctDNA slope). The studies by Tie et al. (2015) and Hsu et al. (2018) also applied quantitative levels of ctDNA to improve clinical utility. The first of these studies showed that ctDNA (mutation) changes from baseline to post-treatment could be measured as an absolute level or a fold change, with the fold change being a better predictor of tumor response. A ≥10 fold-decrease in ctDNA levels from baseline to the end of cycle 1 had an odds ratio (OR) of 5.25 (1.38–19.93) for predicting response to therapy. The study by Hsu et al. (2018) applied a threshold level for ctDNA change. A >80% ctDNA decrease (measured from the highest MAF of the panel at each time point) gave a sensitivity of 100% with a specificity of 71% for treatment response. For a ctDNA decrease <80%, the OR for response was 0.026 (95% CI 0.001–0.637). Despite these promising results they reported that disease progression was not associated with an increasing ctDNA level in all patients. This was most likely because only a 12 gene panel was applied, which may not have been sufficiently sensitive for sub-clonal variants.

In the following sections we will review articles that have investigated the use of ctDNA in monitoring the response to specific systemic treatments. This will be divided into the main treatment categories of standard chemotherapy (cytotoxic agents), epigenetic therapy, targeted therapies including anti-EGFR and anti-VEGF therapies, and other therapies. A summary of the studies that have calculated hazard ratios for progression free survival (PFS) or overall survival (OS) with ctDNA results (or correlation with tumor burden) are included in **Table 2**, with the more descriptive studies summarized in **Supplementary Table 2**.

**Table 2:** Monitoring response to therapy with circulating tumor DNA (ctDNA).

Reference	ctDNA type/analysis method	Sample size	Study type	Treatment	When was blood collected	Stage (number)	Hazard ratio (95% CI) for progression free survival (multivariate)?	Hazard ratio (95% CI) for overall survival (multivariate)?	Correlation with tumor burden
Neoadjuvant chemotherapy									
(Tie et al., 2019)	1 somatic mutation selected from the most prevalent of 15 tumor tissue mutations/PCR with Safe-Seq	159	Prospective	Neoadjuvant chemoradiotherapy	4–6wk post neoadjuvant therapy	II (35) III (124)	ctDNA positive: HR 6.0 (2.2–16.0) (multivariate)	Not reported	Not reported
Chemotherapy									
(Yao et al., 2018)	Mutations in *KRAS, NRAS, HRAS, BRAF* PCR with HiSeq	27	Prospective	Standard chemotherapy	During treatment (but time points not clear)	mCRC	All ctDNA: HR 3.351 (1.17 – 9.58)	Not reported	Not reported
(Tie et al., 2016)	Single mutation per patient, as identified in original tumor/PCR with Safe-Seq	52	Prospective	Standard chemotherapy	4–10wk post operatively 3 monthly for up to 2 years	Stage II	ctDNA immediately post adjuvant chemo: HR 11 (1.8–68)	Not reported	Not reported
(Takayama et al., 2018)	*KRAS* mutations/ddPCR	85	Prospective	Chemotherapy	During treatment (time course not clear)	mCRC	Not reported	Not reported	There was an association in change in ctDNA levels and tumor shrinkage or growth
(Amatu et al., 2016)	Methylated *MGMT*/PCR (MethylBEAMing)	29	Retrospective	Temozolomide	before and during therapy	mCRC with *MGMT* promoter hypermethylation of tumor tissue	Not reported	Not reported	correlation between methylation variation and tumor shrinkage (p = 0.008)
(Barault et al., 2018)	Methylation panel (*EYA4*, *GRIA4*, *ITGA4, MAP3K14-AS1,* *MSC*)/PCR	137 for tumor burden; 29 for longitudinal assessment	Retrospective	Temozolomide	At time of radiological disease in 137; and with follow-up bloods in 29	mCRC	For a decrease in methylation, HR 0.48 (0.17–0.87)	Not reported	Higher methylation was associated with tumor burden
Anti-EGFR therapies									
(Kim et al., 2018)	*KRAS* and *NRAS* mutations *BRAF* and *EGFR* mutation/Plasma*Select*-R 64-gene panel assay (next generation sequencing)	164	Prospective	Panitumumab	30–33 days after last dose of treatment	mCRC *KRAS* WT status (chemorefractory)	Without emergent *RAS* mutations: HR 0.91 (0.65–1.26) Without emergent *BRAF* mutation: HR 0.93 (0.64–1.35) Without emergent *EGFR* mutation: HR 0.65 (0.47–0.91)	Without emergent *RAS* mutations: HR 1.16 (0.81–1.68) Without emergent *BRAF* mutation: HR 1.68 (1.12–2.51) Without emergent *EGFR* mutation: HR 1.24 (0.87–1.78)	Not reported
(Sun et al., 2018a)	*KRAS, NRAS or BRAF* Mutations/ddPCR	140	Prospective	Chemotherapy and cetuximab or panitumumab	Baseline, monthly intervals	mCRC *KRAS/NRAS/* *BRAF* wild type	Not reported	ctDNA detection – poor OS HR 0.88 (0.59–1.33) (multivariate)	Not reported
(Siena et al., 2018)	*RAS* mutations/BEAMing	39	Prospective	Panitumumab + irinotecan	Baseline, during treatment, at progression of disease	mCRC *KRAS* wild type	HR 1.08 (0.49–2.38)	Not reported	ctDNA detectable before radiographic disease progression
(Xu et al., 2017)	genes involved in EGFR signalling (mutations in *AKT1, BRAF, EGFR, KRAS, NRAS, PIK3CA, PTEN, TP53*)/Targeted amplicon ultra-deep sequencing	32	Prospective	Cetuximab +/− chemotherapy	Baseline, every 4 weeks, until progression of disease/last sample	mCRC with acquired cetuximab resistance	*PIK3CA* or *RAS* mutation detection: HR 1.26 (0.79 to 2.01)	Not reported	ctDNA levels in 10 patients correlated with stage of disease (n = 10/20 patients had mutations in the 8 genes)
(Cremolini et al., 2018)	*RAS* and *BRAF* mutations/ddPCR and ultra-deep next-generation sequencing	28	Prospective	Chemotherapy (irinotecan) and cetuximab (rechallenge)	At rechallenge baseline	mCRC with RAS and BRAF wild-type tissue	*RAS* wild type: HR 0.44 (0.18–0.98)	HR 0.58 (0.22–1.52)	Not reported
(Peeters et al., 2019)	Mutations in *KRAS, NRAS, BRAF, MAP2K1, PIK3CA,* and *PTEN*/Next generation sequencing	208	Prospective	panitumumab	At baseline and 30–33 days after finish of treatment	mCRC *KRAS* WT tissue (chemorefractory)	Not reported	*KRAS* mutations P < 0.05	Not reported
Anti-VEGF therapies									
(Vandeputte et al., 2018)	Targeted sequencing1 to 4 mutations personalized from findings in tumor tissue/ddPCR	20	Prospective	Regorafenib	Baseline, day 14 during treatment, every 2 cycles until progression of disease	mCRC refractory to standard therapy	Early increase (D14) in mutated copies/mL HR 6.12 (p = 0.008)	Early increase (D14) in mutated copies/mL HR 8.02 (P = 0.004)	Not reported
(Khan et al., 2018a)	*KRAS* mutation/ddPCR	21	Prospective	Regorafenib	Baseline, 4 weekly until progressive disease	mCRC with *RAS* mutant tissue chemorefractory	Decrease in ctDNA after 8wk HR 0.21 (0.06 to 0.71)	Decrease in ctDNA after 8wk better HR 0.28 (0.07-1.04)	Not reported
(Tabernero et al., 2015)	*KRAS, PIK3CA, BRAF* mutations/BEAMing	337	Retrospective	Regorafenib	After treatment	mCRC chemorefractory	*KRAS* wild type: HR 0.52 (0.35–0.76) *KRAS* mutation: HR 0.51 (0.40–0.65)	*KRAS* wild type: HR 0.67 (0.41–1.08) *KRAS* mutation: HR 0.81 (0.61–1.09)	Not reported
(Thomsen et al., 2018)	Mutations in *KRAS, NRAS, BRAF/*ddPCR	20	Prospective	Chemotherapy and bevacizumab	Before treatment start and at every cycle until progressive disease	mCRC with *RAS/RAF* tumor mutations	HR 0.16 (p = 0.017)	Not reported	Not reported
(Yamauchi et al., 2018)	Mutations (30 were single nucleotide variants and 22 were nucleotide insertions or deletions)/Next generation sequencing	21	Prospective	Chemotherapy and bevacizumab	Baseline, remission, post-progression	mCRC	Not reported	Reduction in MAF associated with better survival: 16.6 vs 32.5mo p < 0.001 MAF at remission: HR 22 (2.5–190) (multivariate)	Positive correlation between MAF and tumor load r = 0.56 (baseline) r = 0.49 (remission) r = 0.75 post progression
(Herbst et al., 2017)	Methylated *HPP1/*Methy-Light PCR	467	Prospective	Chemotherapy and bevacizumab	Baseline and day 15 or 22	mCRC	Not reported	Detection: HR 1.86 (1.37–2.53) Reduction to non-detectable levels post treatment: Pre/post Rx: Pos/neg vs neg/neg HR 1.41 (1.00–2.01) Neg,pos/pos vs neg/neg HR 2.6 (1.86–3.64)	Not reported
Combination therapies									
(Tie et al., 2015)	ctDNA based on presence of mutations in tumor/PCR with Safe-Seq	52	Prospective	First line chemotherapy with or without cetuximab or bevacizumab	Before treatment, 3 days after standard treatment, and after cycle 1	mCRC chemotherapy naïve	≥10 fold-change in ctDNA: HR 1.87 (0.62–5.61)	p > 0.05	Correlation of pre-treatment ctDNA and tumor burden r = 0.50, p < 0.001
(Hsu et al., 2018)	Panel of mutations (*AKT1, BRAF, CDKN2A, CTNNB1, EGFR, HRAS, KRAS, NRAS, IDH1, IDH2, PIK3CA, TP53*)/PCR and ultra-deep next generation sequencing	18 with follow-up	Prospective	FOLFIRI + cetuximab or bevacizumab	Before and after treatment (at weeks 5–13 and 16–28)	mCRC	ctDNA decrease >80% HR 0.22 (0.03–0.59)	Not reported	Correlation of ctDNA decrease with tumor shrinkage: r = 0.551, p = 0.041
(Garlan et al., 2017)	Mutations (*KRAS, BRAF, TP53*) and methylation (WIF1, NPY)/ddPCR	73	Prospective	1^st^ or 2^nd^ line chemotherapy with or without targeted therapy	Before each chemotherapy cycle (week 0, 2 and 4)	mCRC receiving 1^st^ or 2^nd^ line chemotherapy	An increase in ctDNA after 1–2 cycles had poor PFS: HR 3.62 (1.30–10.04) (multivariate)	An increase in ctDNA after 1–2 cycles had no association with OS: HR 2.26 (0.59–8.63) (multivariate)	Not reported
(Shitara et al., 2019)	*RAS, BRAF, EGFR,* mutations; *HER2, and MET* amplification/ddPCR	98	Prospective	Regorafenib then cetuximab +/− irinotecan, or cetuximab +/− irinotecan followed by regorafenib	Baseline, during and after treatment	mCRC with *KRAS* wild type tissue failure of initial chemotherapy	Not reported	Emerging genetic alteration HR 2.02 (p = 0.027) (multivariate)	Not reported
Other therapies									
(Overman et al., 2016)	Methylated vimentin/PCR, next generation sequencing, pyrosequencing	26	Prospective	Azacitidine and capecitabine/oxaliplatin	Baseline, cycle 1 day 5, cycle 2 day 1, cycle 2 day 5, and at each restaging	mCRC refractory to fluoropyrimidine and oxaliplatin therapy	Not reported	Not reported	Baseline methylated vimentin correlated with tumor volume (R = 0.75, p < 0.0001)
(Corcoran et al., 2018)	*BRAF* mutations/BEAMing	85	Prospective	Dabrafenib + panitumumab ± trametinib	Baseline, week 4 of treatment, at disease progression	Advanced or mCRC with BRAFV600E-mutant tissue	Not reported	Not reported	Reduction in ctDNA correlated significantly with the best percentage tumor change (p = 0.001, r = 0.414)
(Siravegna et al., 2018)	73-gene panel assessed. *KRAS, NRAS, BRAF, PIK3CA, MAP2K1, ERBB2, EGFR, MET* mutations and/or copy number alterations/ddPCR	30	Prospective	Trastuzumab & lapatinib	Baseline, every 15d during treatment with HER-2 blockade, at radiological progression or end of treatment	mCRC	Not reported	Not reported	CT size correlates with ctDNA levels; in 1 patient with 8 mets, size on CT correlated with ctDNA levels
(Hong et al., 2016)	*BRAF V600E,* MAPK mutation/ddPCR and next generation sequencing	12	Prospective	Vemurafenib, irinotecan, and cetuximab.	Serial samples	mCRC with BRAFV600E mutant tissue	Not reported	Not reported	ctDNA level correlated with radiographic changes

ctDNA, circulating tumor DNA; ddPCR, droplet digital PCR; HR, hazard ratio; MAF, mutant allele frequency; mCRC, metastatic colorectal cancer; OS, overall survival; PFS, progression free survival; 95% CI, 95% confidence interval.

### Response to Chemotherapy

Seven studies have demonstrated how ctDNA can monitor disease response to cytotoxic agents. This has generally been done by using a panel of biomarkers (rather than a single mutation or methylation marker), with a panel being more informative due to the heterogenous nature of CRC mutations. A panel is particularly relevant in monitoring therapy responses as the tumor can evolve and treatment resistance can develop, causing changes to the mutation and methylation profiles (Saluja et al., 2018). It provides the best opportunity to detect tumor evolution as early as possible. This is supported by a study that compared monitoring of a single mutation in ctDNA with a panel in patients with mCRC undergoing either oxaliplatin or irinotecan based chemotherapy (Yao et al., 2018). *KRAS* was the most common mutation found in ctDNA (present in 32.9% of samples). *BRAF* mutations were present in 2.6% of patients, *HRAS* in 4.0% and *NRAS* in 2.6%. Measurement of only ctDNA *KRAS* mutations was not significantly associated with PFS (p = 0.06), but a positive result with a ctDNA panel (*KRAS, NRAS, HRAS,* or *BRAF* mutations) was a significant indicator for increased risk for disease progression compared with absence of ctDNA (HR 3.4, 95% CI 1.17–9.58). While this supports the notion that the presence of any ctDNA is a measure for poor prognosis and is an indicator for residual disease despite therapy, this study did not report on the quantification of ctDNA, which may have improved the predictive power of their model.

Another study applied a panel of ctDNA biomarkers (selected from tumor tissue analysis) in a patient with mCRC with first-line treatment with FOLFOX and second-line treatment with FOLFIRI (de Figueiredo Barros et al., 2018) and showed that the baseline sample had high frequency of *KRAS* and *TP53* MAF which fell below the detection limit after one month of chemotherapy. A subsequent increase was associated with disease progression and resistance to therapy. The same observations were made in two small studies of mutation ctDNA (n = 7) (Ng et al., 2017) and a panel of mutation and methylation biomarkers (n = 3) (Garrigou et al., 2016) that showed that ctDNA levels became detectable again due to progression of disease.

Of the studies focussing on response to standard chemotherapy, only one applied ctDNA in non-metastatic CRC patients (stage II), using a personalized ctDNA panel (based on tissue mutations) (Tie et al., 2016). Assessment of ctDNA at the end of adjuvant therapy showed that a positive result was predictive of disease recurrence (HR 11, 95% CI 1.8–68).

The use of the alkylating agent temozolomide is not standard practice for CRC, but two studies have assessed the use of methylated ctDNA to monitor response of this agent in patients with mCRC. One study applied a panel of methylation markers (*EYA4*, *GRIA4*, *ITGA4, MAP3K14-AS1,* and *MSC*) (Barault et al., 2018) in 25 patients, and the other measured methylated *MGMT* in 29 patients with MGMT promoter hypermethylation of tumor tissue (Amatu et al., 2016). While both studies showed that ctDNA correlated with tumor changes, only the study using a ctDNA panel reported an association with progression free survival (**Table 2**). So even though ctDNA may correlate with tumor changes, it does not necessarily translate to beneficial clinical outcomes.

### Response to Epigenetic Modifiers

With the search criteria applied in this review, there was only one study that used ctDNA to monitor response to epigenetic therapy. In this phase I trial, 22 patients were administered irinotecan and guadecitabine (30mg/m^2^ or 45mg/m^2^). With treatment, measurement of ctDNA with methylated LINE1 showed a decrease in ctDNA of 6.6% for the lower dose of guadecitabine and 13% for the higher dose (Lee et al., 2018). However, by cycle 2 (day 15) there was little difference in changes to ctDNA between patients with stable and progressive disease, showing limited utility in methylated LINE1 alone to monitor the response to epigenetic modifying agents.

### Targeted Therapy

Targeted therapies are often used in the treatment of patients with mCRC. Below we have described the use of ctDNA in monitoring response to targeted treatments including anti-EGFR and anti-VEGF therapies.

#### Anti-EGFR Therapy

##### Monitoring Response to Therapy

Antibodies specific to EGFR (e.g. cetuximab and panitumumab) are an example of targeted treatment which are used in patients with RAS WT tumors. Many patients, however, invariably experience resistance to anti-EGFR agents and have tumor progression, likely due to clonal evolution under the selective pressure of EGFR inhibition, or the acquisition of new genetic alterations (Zhang et al., 2019). The resistance in 60% of cases is thought to be due to the emergence of *RAS* mutations (Misale et al., 2012) and therefore utilizing ctDNA may help detect resistance early and guide treatment changes accordingly (Klein-Scory et al., 2018).

Many studies were identified that used various ctDNA biomarkers to specifically monitor therapy with anti-EGFR agents. Six of the studies provided calculations of hazard ratios for PFS and OS (**Table 2**), while 18 were mainly descriptive studies (**Supplementary Table 2**). The studies can be further divided into those focussing on monitoring response to therapy, or rechallenge of therapy, and those that used ctDNA to assess the changes that occur with resistance to therapy.

Mutations of genes related to the EGFR signalling pathway, such as in *EGFR, KRAS, BRAF*, and *PIK3CA*, have been measured in studies that have monitored the response to cetuximab and panitumumab in mCRC patients. The measurement of *KRAS* mutations in ctDNA show an increase in level of ctDNA prior to disease progression in a number of small (n < 10) descriptive studies (Yamada et al., 2016; Hosoya et al., 2017; Trojan et al., 2017; Ghatalia et al., 2018; Klein-Scory et al., 2018; Knebel et al., 2019). Another study of 24 *KRAS* WT patients undergoing treatment with chemotherapy and either cetuximab or panitumumab demonstrated that *KRAS* mutations could be detected in ctDNA in the majority of affected patients up to 3 months prior to detection of disease progression with a CT scan (Yamada et al., 2016). ctDNA mutations were not detected in patients who maintained an optimal response to anti-EGFR treatment. Detection of *KRAS* mutations prior to disease progression have also been reported as early as 8 months before detection with radiology (Khan et al., 2018b).

It has also been reported that the loss of *PIK3CA* mutations in ctDNA following cetuximab treatment was associated with stable disease in four patients. The two patients who remained ctDNA positive developed disease progression (Zeng et al., 2017), with a separate case study demonstrating how the ctDNA levels correlated with clinical status of the patient and that they were able to detect new mutations in *KRAS* and *PIK3CA* before clinical progression of disease (Kim et al., 2015). Similarly, Toledo et al. (2017) suggested that rapidly increasing levels of ctDNA were associated with a poor prognosis, and changes to levels of *KRAS, NRAS, BRAF*, and *PIK3CA* mutations in these 25 *KRAS* WT mCRC patients preceded the clinical changes. Patients with no ctDNA had a prolonged response, a small increase in level correlated with resistance to therapy, and an upsurge occurred prior to progression of disease. Other small studies have had similar observations in correlations between ctDNA level and treatment response when also monitoring for EGFR extracellular domain (ECD) variants (Van Emburgh et al., 2016), *MET* amplification (Bardelli et al., 2013), *STK1* (Kastrisiou et al., 2018), and a panel of methylated genes (Barault et al., 2018).

Despite these positive reports for ctDNA, a few studies have also reported a lack of correlation of ctDNA and clinical outcomes with cetuximab or panitumumab treatment. For example, Thomsen et al. (2019) did not find a statistically significant correlation between ctDNA (*RAS, RAF*, and *EGFR* mutations) and response to treatment or disease progression (RR = 1.24 for progression or death, p = 0.25). In addition, in a study of 238 patients with paired plasma samples pre- and post-third line treatment with panitumumab, PFS, and OS were no different between those with and without emergent RAS mutations (Kim et al., 2018). In addition, rates of stable and progressive disease were similar between the two groups. In this study a highly sensitive plasma assay was utilized to detect mutations, detecting as little as 0.1% mutant DNA, which is much higher than some assays which have a limit of detection of 5% mutant DNA. This highly sensitive detection can result in detection of levels of mutations which may not be clinically relevant. This highlights the importance in setting relevant thresholds for ctDNA detection. Another reason for the negative findings in the study by Kim et al. (2018) could be due to other mutations driving the treatment resistance such as *BRAF* and *EGFR*. In this study, the emergence of *BRAF* mutations was significantly associated with shorter OS (HR 1.68), while *EGFR* mutations that developed during treatment were associated with a better PFS (HR 0.65). Yet further studies in the same patients showed that total mutational load (assessed with a 63 gene panel) was inversely associated with OS (Peeters et al., 2019). The main three genes with gains in mutations were *KRAS, BRAF*, and *PIK3CA*. Of 113 patients that had no ctDNA mutations detected at baseline, 54 gained mutations with treatment, showing the importance of monitoring despite negative ctDNA results at baseline.

##### Monitoring Response to Re-Challenge With Anti-EGFR Therapy

Measurement of ctDNA has also been used to monitor third-line treatment, and response to rechallenge with anti-EGFR therapy. A 2014 study of mCRC patients on third line treatment with anti-EGFR noted that the MAF level of *KRAS* decreased during treatment in responding patients and increased at progression of disease (p = 0.008). A decrease in ctDNA of more than 50% was associated with a better response to treatment (p = 0.04). The detection of new mutations in *KRAS* wild type patients, had a mean lead time of 36 days prior to radiographic evidence of progression (n = 5) (Spindler et al., 2014).

A study has also demonstrated how ctDNA *KRAS* mutation load can explain efficacy of re-challenging patients with anti-EGFR therapy after a period of withdrawal (Siravegna et al., 2015). The *KRAS* mutation load in ctDNA declined when anti-EGFR therapy was withdrawn. Two patients were rechallenged with anti-EGFR therapy, and these patients exhibited pulsatile levels of *KRAS* MAF. This demonstrates that the clonal redistribution occurs dynamically in CRC patients. Cremolini et al. (2018) also studied the response of patients to re-challenge with cetuximab following the initial development of resistance to this treatment. Metastatic CRC patients whose tumors were *KRAS/NRAS/BRAF* WT and were initially responsive to cetuximab subsequently developed resistance. Following disease progression, these patients were rechallenged with the targeted treatment. ctDNA measurements were performed at the time of rechallenge. Patients who were wild-type had a better progression-free survival than those who had ctDNA mutations (HR 0.44; 95% CI, 0.18–0.98).

Two studies have investigated ctDNA monitoring in re-challenge with anti-EGFR, using antibodies that bind to multiple regions of EGFR. Montagut et al. (2018) investigated ctDNA levels in patients receiving Sym004. No clinical correlation was found between detection of ctDNA *RAS, BRAF,* and *EGFR* (ECD) mutations and treatment response. The results revealed a high interpatient heterogeneity following therapy, further supporting the idea of clonal tumor evolution during treatment. The other study monitored the response to MM-151 in anti-EGFR refractory mCRC patients harboring EGFR ECD mutations. During treatment, the ctDNA MAF decreased with treatment and increased at progression. Serial monitoring was performed in only 2 patients however, and more data is needed to validate the utility of ctDNA in this area (Arena et al., 2016).

##### Monitoring the Development of Resistance

In patients who have acquired resistance to anti-EGFR therapy, retrospective studies have identified important genetic alterations such as *MET/HER2* amplification (Raghav et al., 2016; Takegawa et al., 2016), *KRAS* mutations (Morelli et al., 2015; Tabernero et al., 2015) and other *MAPK* gene mutations (Bettegowda et al., 2014; Xu et al., 2017) that are associated with the resistance to therapy. A study compared digital droplet polymerase chain reaction to next-generation sequencing (NGS) and found that NGS enabled detection and monitoring of genetic alterations in a broader genome region rather than a targeted site. This technique may enable mechanisms of acquired resistance to be found early, allowing interventions for prevention or reversal of resistance (Zhang et al., 2019). One such study performed genome-wide analyses of ctDNA in one patient and found *KRAS* mutations and *MET* amplification at the emergence of anti-EGFR resistance (Diaz et al., 2013). Another study reported that *MET* amplification was detected in 22.6% of patients who developed resistance to anti-EGFR therapy (Raghav et al., 2016) and there were similar percentages with *HER2* amplification (22% patients) (Takegawa et al., 2016). Although there is a low prevalence of *HER2/MET* amplification in mCRC, it is higher in patients with acquired resistance and thus it could be incorporated into a panel of biomarkers for detecting resistance (Raghav et al., 2016).

This is supported by a study from Italy that found that *KRAS* mutations and *HER2/MET* amplifications were the most common resistance mechanisms detected in ctDNA and tumor tissue analysis, and that patients with *MET* amplification had a shorter PFS (Pietrantonio et al., 2017). Analysis of pre-treatment tumor tissue revealed higher genetic heterogeneity in patients who developed resistance, emphasising the idea of clonal evolution and selective pressure from anti-EGFR therapy (Pietrantonio et al., 2017).

Other studies have reported new *KRAS*, *EGFR,* and *PIK3CA* mutations in ctDNA with acquired anti-EGFR resistance. A 2014 study reported >1 emergent mutations in MAPK pathways in 96% of patients with acquired anti-EGFR resistance. Mutations at codon 61 of *NRAS* and *KRAS* represented 46% of detected mutations, which highlights the importance of *RAS* mutations as a key mechanism of resistance (Bettegowda et al., 2014). Similar proportions were reported in other studies of 44% (of n = 62) (Morelli et al., 2015) and 48% (of n = 86) with *KRAS* mutations detected in ctDNA (Tabernero et al., 2015), and 8% with *EGFR* mutations (of n = 62) (Morelli et al., 2015). Detection of both *EGFR* and *KRAS* mutations in some patients highlights that multiple resistance mechanisms can simultaneously occur. A case report also investigated *KRAS* and *EGFR* mutations in ctDNA and found that *KRAS* mutations emerged earlier than *EGFR* ECD variants, and that *KRAS* mutations were associated with a shorter PFS. It was suggested that these mutations may be occurring independently in different tumor clones (Van Emburgh et al., 2016). Mutations in the MAPK pathway have been shown to be present in the absence of *RAS* mutations (Bettegowda et al., 2014), which suggests that these mutations should also be tested when considering anti-EGFR treatment.

However, there are mixed reports on whether the emergence of mutations correlates with resistance to anti-EGFR treatment. Some studies report that emergence of mutations in ctDNA do not immediately correlate with clinical resistance, and that there is a lag time to progression of disease, but this may also be a reflection of the sensitivity of ctDNA over radiological imaging in detecting disease progression. Xu et al. (2017) found that the detection of *PIK3CA* (22%) or *RAS* (25%) was not associated with a statistically significant change in PFS (HR 1.26; 95% CI 0.79 to 2.01; P = 0.34). A study by Siena et al. (2018) found that median PFS was similar between patients who developed *KRAS* mutations and those who remained WT at progression (HR = 1.08 95% CI 0.49–2.38 p = 0.84). However, a larger study of 140 patients revealed that detection of ctDNA (*KRAS, NRAS* and *BRAF* mutations) during anti-EGFR therapy was associated with poorer OS (HR 0.88 95%CI 0.59–1.33 p = 0.088) (Sun et al., 2018a).

#### Anti-VEGF Treatment

Vascular endothelial growth factor receptor (VEGF) is a signalling protein involved in the production of new blood vessels and mutations can result in cancers developing the ability to produce unchecked angiogenesis thereby promoting tumor growth (Ellis et al, 2000). Therapies that block the VEGF receptor can intercept this pathway thereby reducing tumor growth. Available agents for the treatment of a number of cancers including mCRC include bevacizumab (monoclonal antibody to the VEGF-A receptor), ramucirumab (anti-VEGF-R2 monoclonal antibody), ziv-aflibercept (VEGF inhibitor), apatinib (anti-VEGF-R2) and regorafenib (VEGFR2-TIE2 inhibition). A single descriptive study utilized ctDNA for monitoring treatment of CRC with anti-VEGF as a single agent, demonstrating a rising ctDNA preceding radiological recurrence (Chen et al., 2019). More commonly, anti-VEGF treatments are used in the setting of metastatic disease as part of combination chemotherapy, so it can be difficult to extrapolate on the effectiveness of ctDNA as a monitoring tool for the effectiveness of anti-VEGF treatments, but the studies using targeted treatment (monoclonal antibodies or with small molecular kinase inhibitors) are described below.

There is currently no test for detecting who will respond to anti-VEGF therapy. The descriptive study by Chen et al. therefore used a panel of 1,201 markers to attempt to identify which mutations may predict improved survival in mCRC patients receiving apatinib (Chen et al., 2019). With further research, there is the potential for ctDNA mutation testing for VEGF mutations to enable selection of patients who would be suitable for anti-VEGF therapies, in the same way as for anti-EGFR therapy. An *in vitro* study using wild-type and mutated VEGF cell lines showed that the cells with mutations were resistant to anti-VEGF treatments. The single stage IV CRC patient in this study was also shown to have no mutations in the *RAS* pathway, but was found to have a VEGFR2 mutation on ctDNA and was resistant to bevacizumab-containing chemotherapy (Toledo et al., 2018).

Of a further five studies that used ctDNA to monitor response to treatment of mCRC patients with chemotherapy with bevacizumab, assays for mutations and methylation have been performed, with all showing utility in reflecting progression of disease. The studies measuring *RAS* and/or *BRAF* mutations have also demonstrated that increased ctDNA levels precede CT detection of disease progression. Following the initial decrease in ctDNA levels with treatment, increased ctDNA levels could detect disease progression a few weeks (n = 2) (Alcaide et al., 2019) to 51 days earlier (n = 77) (Thomsen et al., 2018) than the CT scan. A high ctDNA level at any time during treatment was strongly associated with progression of disease (RR = 4.58, 95%CI = 1.99–10.51, P < 0.001) (Thomsen et al., 2018). In a small observational study (n = 3) it could also be seen that 3–5 new mutations were observed at disease progression (Vietsch et al., 2018), which highlights the benefits of applying a large panel when measuring mutations in ctDNA.

One such study assessed changes in ctDNA MAF from a panel of 52 mutations (Yamauchi et al., 2018). The decrease in MAF matched clinical response, however as treatment resistance developed, MAF increased. If the MAF reduced to below the median level in the remission period, there was improved survival (32.5 months vs 16.6 months for high MAF, p < 0.001). Low MAF at remission (< 2%) was an independent predictor of OS (HR 22; 95% CI 2.5–190).

In a study that measured ctDNA methylated HPP1 levels, a statistically significant decrease in ctDNA compared to pre-treatment was observed following induction chemotherapy (n = 337, p < 0.0010). Overall survival was better for those who became negative post-treatment, compared to those who were positive post treatment (regardless of their initial status), suggesting that the drop in ctDNA was a surrogate marker for treatment response. Overall survival for patients who became negative was lower than for patients who were negative on both pre- and post-treatment samples (HR 1.41, 95% CI 1.86–3.64), indicating that those who responded to treatment fared better than those who either had false negative results or whose tumors did not express the mutations that were tested (Herbst et al., 2017).

A number of papers have discussed the multikinase VEGF inhibitor regorafenib and the role of ctDNA (specifically KRAS mutations), in monitoring treatment response. As in the studies that monitored treatment with bevacizumab, Khan et al. found that the fractional abundance of *KRAS* mutations decreased with response and increased with resistance to regorafenib treatment. An early and sustained reduction in ctDNA was associated with a longer PFS (HR 0.21, 95% CI 0.06 to 0.71) and OS (HR 0.28, 95% CI 0.07–1.04) (Khan et al., 2018a). Another study reported similar results using targeted sequencing of 47 CRC-associated genes. There was an initial decrease in ctDNA level, then an increase at disease progression. An early increase in ctDNA was associated with a shorter PFS (HR 6.12, P = 0.008) and OS (HR 8.02, P = 0.004) (Vandeputte et al., 2018). Similar findings were made by Tabernero et al. who also noted that detection of *KRAS* mutations in ctDNA was associated with worse PFS and OS with regorafenib treatment (Tabernero et al., 2015). One study that applied a panel of ctDNA mutations (*KRAS, PIK3CA,* and *BRAF*) showed that an early reduction in mutant ctDNA correlated with a longer PFS. Serial sampling was important for this result as they found that baseline ctDNA level was not predictive of PFS (Wong et al., 2015). These studies all demonstrate the utility of ctDNA in assessing response to regorafenib, however insufficient detail has been provided in the latter study to determine whether the mutations other than *KRAS* are beneficial in the ctDNA panel (Wong et al., 2015).

#### Combination Treatments

A few studies have assessed anti-EGFR therapy in combination with agents other than standard chemotherapy. Hong et al. monitored ctDNA levels in BRAFV600E mutant mCRC patients while on combined anti-EGFR and BRAF inhibitors. The ctDNA level correlated with radiographic changes and new mutations in MAPK genes were detected at disease progression (Hong et al., 2016). Similar to a previous study (Spindler et al., 2014), the proportion of ctDNA reduction correlated with response to treatment (Spindler et al., 2014; Hong et al., 2016). Similar results were observed in a study from 2018 where BRAFV600E mutant mCRC patients were treated with anti-EGFR therapy, BRAF inhibitor and a MEK inhibitor (Corcoran et al., 2018). The level of ctDNA *BRAF* mutations correlated with clinical response to treatment, with greater reduction in responding patients compared to those with stable or progressive disease (p = 0.004) and was correlated to percentage tumor change (p = 0.001, R = 0.414). They also noted rebound increases in ctDNA *BRAF* mutations in patients who initially responded and then had disease progression. Additionally, 48% of the patients had emergence of new *KRAS* and *NRAS* mutations at disease progression, showcasing tumor heterogeneity (Corcoran et al., 2018). Both of these studies suggest utility of testing ctDNA *BRAF* levels in patients receiving combination therapy of anti-EGFR and BRAF inhibitor to monitor tumor response.

Two studies have monitored MET copy number variation (CNV) in ctDNA to determine response to treatment with a MET amplification inhibitor. The first case report trialled a treatment of cabozantinib (MET amplification inhibitor) and panitumumab (anti-EGFR therapy) in an anti-EGFR refractory mCRC patient with MET amplification. Results showed that MET inhibition restored the sensitivity to anti-EGFR in a synergistic manner. The level of ctDNA (MET amplification and *KRAS* mutants) correlated with tumor burden and response to therapy (Jia et al., 2018). The second case report trialled treatment of crizotinib (MET amplification inhibitor) and vemurafenib (BRAF inhibitor). They found an initial decrease in *BRAF* mutations and MET CNV, followed by an increase in *BRAF* mutations compared to baseline, and an increase in MET CNV at progression of disease (Oddo et al., 2017).

Another case report of an anti-EGFR refractory mCRC patient investigated MEK1 inhibitor (trametinib) and anti-EGFR (panitumumab) combination therapy. It was shown that the ctDNA MEK1 level declined initially with radiological response; however there was persistent *KRAS* mutant elevation, which was later found to be related to non-responding metastases. This report highlights intra-patient tumor heterogeneity and the use of ctDNA to gain a more comprehensive genomic picture of the tumor (Russo et al., 2016b).

Two studies have demonstrated that ctDNA can also monitor response to a combination of anti-EGFR and regorafenib treatment. Shitara et al. (2019) found that 17/98 patients had >1 genetic alteration in ctDNA *RAS/BRAF* mutations post-therapy, and this was associated with shorter overall survival. The other study showed that in a cohort of 11 patients, ctDNA *KRAS* mutations emerged in 3 of these patients prior to radiographic disease progression. During subsequent regorafenib therapy *KRAS* mutations disappeared in 2 out of these 3 patients with detectable *KRAS* at the start of treatment. These patients were then re-challenged with anti-EGFR antibody and one of them developed *KRAS* mutations. The results showcase the dynamic clonal evolution during treatment and use of regorafenib in resensitizing patients with acquired *KRAS* mutations (Kakizawa et al., 2017).

#### Other Treatments

Other drug-based approaches have been investigated for patients who are refractory to standard chemotherapy, including checkpoint blockade, tyrosine kinase inhibitors (other than EGFR and VEGF), anti-PD1 ALK inhibitors and targeted therapy against the HER2 receptor. As with the therapeutic options described above, ctDNA can also be used to sensitively monitor the effectiveness of these newer therapies and to detect resistance.

Oncogenic activation of TRK receptors leads to downstream activation of the MAPK and AKT downstream pathways and TRK inhibition may decrease cellular proliferation in some patients (Russo et al., 2016a). In a phase Ib/II trial of FOLFOX and dasatinib, with or without cetuximab, 98% of patients receiving all three therapies developed a ctDNA mutation in *RAS* or *BRAF* which may have caused resistance to treatment (Thierry et al., 2017). In those that had *KRAS* mutant status at baseline, 50% developed new mutations at resistance. The results suggest that treatment pressure drives convergent evolution of tumor clones, which leads to treatment resistance.

A case report investigated the use of entrectinib (a pan TRK inhibitor) in a patient with mCRC with an LMNA-NTRK1 rearrangement (which activates TRK receptors). During treatment, the patient initially responded but then became resistant. The ctDNA profile revealed emergence of new NTRK1 mutations which continued to increase in frequency and peaked when disease progression was radiologically evident (Russo et al., 2016a).

Response to immune checkpoint blockade with pembrolizumab (anti–PD-1) was reported in another case study. Measurements of ctDNA for *KRAS* mutations were able to show response to therapy. The baseline MAF decreased from 23% to 2.4% after 2 weeks of treatment and was undetectable from 9 weeks onwards (Ree et al., 2019). The patient had ongoing partial response to treatment for 20 months (at time of publication).

Two studies by Siravegna et al. (Siravegna et al., 2018; Siravegna et al., 2019) monitored patients on treatment that targeted the HER2 receptor (trastuzumab and lapatinib). They were largely descriptive studies, however they reported that ctDNA could be used to identify specific gene mutations and *ERBB2* copy numbers, with the response to therapy, as well as how gene mutations changed during treatment (thereby implicating these genes in medication resistance pathways).

While most studies assess somatic mutations and/or methylation changes for detecting ctDNA, one manuscript has reported a case study of monitoring a genetic rearrangement, which was also detected in the tumor. The patient had multiple metastases from CRC and was treated with an ALK inhibitor. Blood was assayed for the CAD-ALK gene fusion and a *TP53* mutation (Siravegna et al., 2017b), showing that ALK MAF increased with disease progression.

### Summary

While ctDNA can be personalized to monitor response to a certain treatment type if the correct biomarker is used (e.g. monitoring the appearance of *RAS* mutations in response to anti-EGFR treatment, where measuring genetic markers is essential), it can also be used for ultrasensitive monitoring of tumor burden where biomarker selection is less important and is very easily achieved with simpler technology (e.g., utilizing epigenetic biomarkers).

The majority of the studies described have illustrated the use of ctDNA in response to anti-EGFR treatment through serial blood sampling. *KRAS* mutant CRC does not respond to anti-EGFR therapy, so early detection of change in mutation status through ctDNA can personalize treatment schedules. The increase in ctDNA can also be used to prepone radiographic imaging to reassess tumor burden and modify therapy accordingly. The most prevalent resistance mechanism varies between patients and ctDNA studies have demonstrated that besides *KRAS*, other proposed mechanisms of resistance to anti-EGFR therapy include *NRAS, BRAF*, and *EGFR* mutations, HER2 or MET gene amplification and other genes involved in the MAPK pathway.

Detection of resistance, ideally soon after it develops, can be achieved with regular blood sampling, with studies showing that progression of disease through elevated ctDNA levels can be detected months prior to CT detection. It has been suggested that sampling should be performed at least every two months to detect the development of resistance to treatment (Khan et al., 2018b). However, in regards to treatment response, it is important to keep in mind that reduction of tumor burden can be slow, with one study reporting that there were no significant changes between ctDNA levels between baseline and day 3 post-treatment (Tie et al., 2015). Other studies report rapid changes which could be related to the systemic therapy administered. Applying thresholds based on quantitative levels or on slopes of change in levels, have the potential to further improve the sensitivity of ctDNA for use in clinical practice. A recent study has shown that while the use of anti-EGFR therapies has increased in Australia in recent years, the use in patients with *KRAS* WT left-sided tumors in 2017 was only 37% (Semira et al., 2019). The use of ctDNA for determining appropriateness of this treatment should increase confidence in the recommendation of this therapy for eligible patients.

## Neoadjuvant Therapy

As ctDNA shows promise in monitoring adjuvant therapy, it should also have the potential for monitoring patient response to neoadjuvant therapy provided to rectal cancer patients prior to surgery. Bhangu et al. (2018) conducted a retrospective analysis of blood assays performed on samples collected before and after each cycle of neoadjuvant chemotherapy in 34 patients with liver metastases. Four methylation markers (*BOLL, SEPT9, DCC,* and *SFRP2*) were selected from a panel of 48 CRC-associated genes. Patients were categorized as responders or non-responders to therapy, as well as those with progressive disease. *SEPT9* and *DCC* appeared to be the best predictors when assessed before and after each cycle of treatment, correlating with the histologic response. Prospective studies are warranted to corroborate the findings in this retrospective study.

A similar study was performed by Tie et al. (2019) in stage II–III rectal cancer patients, to determine whether the presence of ctDNA was prognostic for the risk of recurrence following chemoradiotherapy and surgery. A single somatic mutation for ctDNA analysis was selected for each patient from mutation analysis of tumor tissue, with blood samples collected 4–6 weeks after chemoradiotherapy. In 144 eligible patients, it was found that with adjustment for gender, stage, CEA levels and the use of adjuvant chemotherapy, a positive ctDNA post-neoadjuvant therapy was highly predictive for disease recurrence (HR 6.0, 95% CI 2.2–16.0).

These two studies further support the use of ctDNA for treatment monitoring. Analysis of ctDNA post-neoadjuvant therapy could be applied in two different ways: to determine the tumor response and suitability for resection, or to determine if it is safe to follow a “watch and wait” surveillance protocol in those with no residual disease rather than proceeding immediately to surgery (Sposato et al., 2018).

## Comparison of ctDNA With CEA

The only blood test currently in clinical use for monitoring of CRC patients post-resection is CEA. While many studies report that it has low sensitivity [reviewed in (Shinkins et al., 2017)], it remains a valid comparator for new ctDNA assays. Several of the described studies of ctDNA biomarkers have also measured CEA levels in response to treatment. These are summarized in **Table 3**. Study outcomes are mixed, with some reporting a good correlation between ctDNA and CEA with responses to therapy [e.g., one study reported a significant correlation coefficient of r = 0.33 (Takayama et al., 2018)], while others report no significant relationship (Tie et al., 2015; Hsu et al., 2018). Similarly, studies that assessed the relationship between CEA and ctDNA prior to treatment had variable findings. One study (n = 128) found no relationship between ctDNA (*PIK3CA* mutation) and CEA positivity (p = 0.414) (Zeng et al., 2017), while another reported a non-significant correlation trend between CEA and higher methylation levels (p = 0.11) in 137 patients with mCRC (Barault et al., 2018). A third study reported a significant correlation between methylated ctDNA and CEA prior to surgery in 184 patients of all CRC stages (methylated *SEPT9* r = 0.270, p = 0.001; methylated *SHOX2* r = 0.313 p < 0.001) (Bergheim et al., 2018).

**Table 3:** Comparison of circulating tumor DNA (ctDNA) and carcinoembryonic antigen (CEA) blood tests.

Monitoring response to treatment				
Reference	Biomarker/analysis method	Cohort details	Comparison of ctDNA with CEA	Comparison of ctDNA and CEA for lead time of detection
Studies that reported discordant findings between CEA and ctDNA				
(Yamauchi et al., 2018)	Gene mutations personalized based on 90 oncogenes tested from tumor tissue/Next generation sequencing	mCRC (n = 21) undergoing treatment with bevacizumab	There was no significant correlation between mutant allele frequency and CEA (r = 0.0082)	Not reported
(Tie et al., 2015)	ctDNA personalized based on presence of mutations in tumor/PCR with Safe-Seq	mCRC (n = 52), receiving 1^st^-line oxaliplatin or irinotecan +/− biological therapy	Changes to ctDNA were predictive of treatment response, but changes to CEA were not predictive	Not reported
(Bhangu et al., 2018)	Panel of 48 methylated genes/PCR	mCRC (n = 34) undergoing systemic neoadjuvant chemotherapy	After 2 cycles of chemotherapy, methylation levels decreased in all patients, but CEA levels were mostly unchanged	Not reported
(Herbst et al., 2017)	Methylated *HPP1/*Methy-Light PCR	mCRC (n = 467) on combination therapy (a fluoropyrimidine, oxaliplatin and bevacizumab)	2–3wk after treatment, hazard ratio for disease progression with a positive ctDNA was higher than for CEA (HR 2.13 vs 1.75)	Not reported
(Corcoran et al., 2018)	*BRAF* mutation/BEAMing	mCRC (n = 85) with BRAFV600E-mutant CRC treated with BRAF inhibitor dabrafenib + panitumumab ± MEK inhibitor trametinib	The change in CEA levels by 6 weeks of treatment was not significantly different between patients with a complete or partial response and stable or progressive disease, whereas a consistent increase in ctDNA was observed at disease progression.	Not reported
(Xu et al., 2017)	8 genes involved in EGFR signalling/Targeted amplicon ultra-deep sequencing	mCRC (n = 10) with acquired cetuximab resistance mCRC and ctDNA mutations	ctDNA increased with treatment resistance in 10 patients; less had an increase in CEA (n = 4 or 5)	Not reported
(Hsu et al., 2018)	12 gene panel of somatic mutations (*AKT1, BRAF, CDKN2A, CTNNB1, EGFR, HRAS, KRAS, NRAS, IDH1, IDH2, PIK3CA, TP53*)/PCR and ultra-deep next generation sequencing	mCRC (n = 18) receiving FOLFIRI + cetuximab or bevacizumab	No correlation between the decrease of ctDNA and CEA levels with treatment (r = 0416, p = 0.232)	Not reported
Studies that reported a trend or significant correlation between CEA and ctDNA				
(Sato et al., 2016)	50 cancer associated genes tested, 24 identified marker mutations selected/Targeted deep sequencing and ddPCR	Stage I-III (n = 28) who underwent resection (28 of 31 patients used in CEA analysis: stage I n = 8, stage II n = 6, stage III n = 17)	CEA correlated with drop in ctDNA levels	Not reported
(Diehl et al., 2008)	Mutations personalized to the tumor tissue (including mutations in APC, KRA, TP53, PIKC3A)/PCR (BEAMing)	Stage II (n = 1), stage III (n = 1) and stage IV (n = 16) who underwent resection	After surgery ctDNA reduced by median of 99% compared to only 32.5% for CEA (p < 0.001) There was a correlation between CEA and ctDNA (R^2^^=^ 0.20 p < 0.001)	Not reported
(Takayama et al., 2018)	KRAS mutations/ddPCR	mCRC (n = 23) receiving chemotherapy +/− biological therapy	There was a significant correlation between ctDNA and CEA for those with tissue KRAS mutations (r = 0.53, p < 0.01) and wild type (r = 0.33, p < 0.01)	Not reported
(Osumi et al., 2019)	14 genes commonly mutated in CRC/Next generation sequencing	mCRC (n = 101) after standard chemotherapy +/− biological therapy	CEA significantly associated with ctDNA levels in patients with metastatic CRC (p = 0.000007)	Not reported
(Ghatalia et al., 2018)	Gene abnormalities (including single nucleotide variants, indels, copy number alterations and, fusions)/Next generation sequencing	mCRC (n = 33) who received 2 lines of therapy	ctDNA levels correlated with CEA (Kendall’s Tau = 0.436, p = 0.001)	Not reported
(Scholer et al., 2017)	Panel of markers including *KRAS, BRAF* mutations/ddPCR	14 stage I–III cases with CRC recurrence after treatment	ctDNA was elevated in 100% of patients prior to relapse (14/14), while CEA was only elevated in 57% of cases (8/14)	ctDNA preceded radiological recurrence significantly earlier than CEA (9.4 months vs 3.3 months, p = 0.02)
(Ng et al., 2017)	Personalized patient specific assays (up to 15 somatic variants per patient)/PCR and next generation sequencing	13 patients (stage I–III n = 10, stage IV n = 3) undergoing 15 surgeries with CRC recurrence after treatment	73% (11/15) cases had an increase in ctDNA prior to recurrence compared to 53% (8/15) that had an increase in CEA.	In one patient ctDNA preceded radiological recurrence by up to 255 days, while CEA was only positive at the time of recurrence.
(Tie et al., 2016)	Single mutation (with the highest mutant allele frequency) in each patient, as identified in original tumor/PCR with Safe-Seq	27 stage II cases with CRC recurrence after treatment	ctDNA more likely to be positive than CEA at time of radiological recurrence (85% vs 41% p = 0.002)	ctDNA preceded radiological recurrence significantly earlier than CEA (167 days vs 61 days, p = 0.04)
(Young et al., 2016)	Panel of methylation markers (*BCAT1, IKZF1)/*PCR	28 patients with CRC recurrence after treatment (stage II n = 17, stage III n = 17, stage IV n = 3)	68% of recurrence cases ctDNA positive, compared to 32% that were positive for CEA (p = 0.002)	Not reported
(Reinert et al., 2016)	2–6 tumor specific assays per patient (personalized)/ddPCR and next generation sequencing	6 patients (stage I n = 1, stage II n = 1, stage III n = 2, stage IV n = 2) with CRC recurrence after treatment	CEA had a higher sensitivity for detecting recurrence compared to CEA (100% vs 67%)	ctDNA preceded radiological recurrence significantly earlier than CEA (10.0 months vs 3.5 months, p = 0.037)

CEA, carcinoembryonic antigen; ctDNA, circulating tumor DNA; ddPCR, droplet digital PCR; mCRC, metastatic colorectal cancer.

While there may be a correlation between ctDNA and CEA measurements, comparison of the sensitivity of ctDNA and CEA for CRC recurrence has consistently demonstrated superiority of ctDNA assays, regardless of biomarker applied (**Table 3**). This could be seen when blood was assayed post-operatively, with one study showing that 79% (11/14) of the patients who had post-operative ctDNA detected subsequently developed disease recurrence compared to 29% with elevated CEA (Tie et al., 2016). The same study showed that ctDNA was more likely to be positive than CEA at the time of radiological recurrence (85% vs 41% p = 0.002). Similar findings have been demonstrated using epigenetic markers (methylated ctDNA *BCAT1* and *IKZF1*), although the sensitivity appears to be slightly lower than for genetic markers, having a sensitivity of 68% compared to only 32% for CEA (Young et al., 2016). Better sensitivity of ctDNA (somatic mutations) compared to CEA has been reported in all studies for recurrence after treatment, with sensitivities reported for ctDNA of 73-100%, with CEA sensitivities ranging from 41-67% (**Table 3**). Monitoring for treatment success with ctDNA also allows for earlier detection of disease recurrence, with ctDNA detecting recurrence 5-10 months prior to CT (Reinert et al., 2016; Tie et al., 2016; Scholer et al., 2017). This is much earlier than the 2-3 month median lead-time for CEA (Reinert et al., 2016; Tie et al., 2016; Scholer et al., 2017) (**Table 3**).

## Discussion

The aim of our review was to assess the different applications of ctDNA for the purpose of monitoring response to treatment for CRC and detection of recurrence. The former can be done by monitoring for residual disease after initial treatment, identifying the appropriateness of treatment type, as well as the response to and resistance to therapy. Assaying of blood for tumor-specific DNA has been shown to allow for serial non-invasive testing and real-time monitoring of treatment efficacy and response. An additional advantage is that ctDNA can provide information on the genetic profile of the tumor at a given point in time, rather than a profile obtained from a small tissue biopsy taken at diagnosis and subject to sampling error. ctDNA might also indicate the presence of micrometastases prior to it becoming evident by imaging. The result is the potential for ctDNA to be ulitized for guiding treatment decisions—initiating, altering, and ceasing treatments, or prompting investigation into the potential for residual disease.

An important consideration is whether or not the early detection of recurrent or metastatic disease using ctDNA for surveillance improves survival. As mentioned previously, the early identification of recurrent disease improves survival by leading to an increase in the rate of operable disease. While it may be used to guide the initiation of systemic therapies, the use of local therapies will be limited, for until a recurrence can be localized, surgery or radiotherapy will not be able to be utilized. Perhaps early treatment of microscopic recurrence with systemic therapy will negate the need for local treatments if they are prevented from progressing, or at least reserve these treatments for resistant disease. Whether or not the identification of CT-occult metastatic disease improves survival in a similar way to imaging-apparent disease remains to be seen, but with ultrasensitive detection of ctDNA there is the ability to detect resistance to therapy, or residual disease as early as possible. In addition, there is the ability to measure the level of ctDNA rather than to express results in a “present or absent” fashion, which will improve clinical application, reflect tumor burden, and allow the reporting of whether the disease is regressing, stable or progressing. New technologies allow for highly sensitive detection of ctDNA (e.g., detection of 1 mutant molecule in 100,000 genome equivalents) (Kidess et al., 2015). With this ultrasensitive level of detection, emerging mutations should not necessarily be acted on immediately by changing therapy. Thresholds need to be established to guide clinical recommendations.

The timing of collection of blood for ctDNA testing must also be considered. Changes to tumor biomarkers have been observed during surgery, with intraoperative high circulating cell free DNA (cfDNA) levels presumably resulting from tumor manipulation (Bhangu et al., 2017). These levels remain elevated for 5 days post-operatively demonstrating the importance of timing of blood collection if the aim is to determine adequate tumor clearance. The study by Diehl et al. estimated that the half-life of ctDNA was 114 mins and proposed that although levels can be dramatically lower within 2 days of surgery, they continue to decrease with increasing time from surgery (Diehl et al., 2008). To be able to apply the ctDNA test for clinical use, clear recommendations for timing of blood collections should be made.

The ability of ctDNA to provide a complete picture of the heterogenous gene mutations present within an individual (reflecting the total of all the different subclones), avoids the potential sampling error associated with tumor biopsy. However, a potential disadvantage of targeting treatments using this method is that in the setting of widespread metastatic disease, the targeted treatments are only likely to be effective for the subclones that harbor the specific mutation against which they act, while other subclones may progress simultaneously. Again, whether ctDNA utilized for this purpose will result in improved survival outcomes is unclear.

One limitation of this review is the lack of standardization of ctDNA detection methods between studies. As can be seen in the Tables, assay methods include PCR, droplet digital PCR, BEAMing, and NGS [details on these technologies have been reviewed previously (Khakoo et al., 2018; Moati et al., 2018)]. These methods can vary greatly in cost, and not all technologies are available in standard laboratories. For standardization it is also important to establish quality controls to account for the inter-laboratory variability. A recent study showed that through sending ctDNA samples to 32 laboratories across Europe for mutation testing, and using six different cell free DNA extraction methods and five different analysis methods, it led to a percentage of errors that could have had implications for clinical decision-making around therapy of 20.1% (Keppens et al., 2018). It has also been shown that artefactual *KRAS* mutations may occur, depending on the method applied (Mariani et al., 2018). Setting a threshold for the MAF may reduce the incidence of false results. Similarly, ultrasensitive techniques should also be applied, otherwise the absence of detectable ctDNA may indicate a poor quality sample or analysis, rather than the absence of disease. A further limitation of the studies is that very few established whether ctDNA was an independent predictor for treatment response. Of 20 studies that calculated a hazard ratio of ctDNA for assessing either PFS or OS, only five studies performed multivariate analysis, correcting for other important variables that may affect survival.

The heterogeneity in ctDNA detection methods is mirrored by the variability in specific genes or gene panels which are selected for testing. Some laboratories measure tumor DNA hypermethylation (epigenetic markers), some focus on a single or handful of gene mutations, while others perform more extensive testing for a much larger number of mutations, and yet others use the detection of a mutation within tumor biopsy to guide which genes are subsequently used for monitoring, creating personalized assays. Tailoring an assay to individual patients based on known cancer mutations enables streamlined subsequent ctDNA testing, however it does not allow for the detection of new mutations and so may not be useful for targeted therapies in advanced disease. Methylation markers can be used without prior knowledge of individual gene variants. Monitoring of responses to any therapy with ctDNA is reliant on the selection of appropriate ctDNA biomarkers. It seems likely that not all ctDNA biomarkers will be equally useful for detecting recurrence, targeting therapy and monitoring tumor burden and we are yet to identify which are best for particular clinical scenarios. This also limits the widespread application and generalisability of these studies to other areas of the world, where other gene markers may be under investigation. In the future, an assay that is able to test for a vast array of gene mutations and gene hypermethylation may be the most effective way of maximizing the benefit of ctDNA and enable some consistency worldwide.

In a recent review article, other factors limiting the clinical utility of ctDNA have been highlighted (Normanno et al., 2018). They report that the main limitations for common application of liquid biopsies include the small sample sizes in most studies and the fact that survival benefits are not demonstrated. While most studies report that their biomarkers of interest can monitor responses to therapy, they have not applied the tumor biomarkers to guide clinical decisions or to carefully define the context in which they are to be applied. A reason for hesitation in the context of surveillance for recurrence or assessment for residual disease is due to the lack of correlation between the first appearance of ctDNA and the first radiological appearance of disease. By delaying treatment until metastatic disease is radiologically confirmed, the opportunity for early treatment and improved survival may be lost. There is at least one study currently underway that has taken the next step in the application of ctDNA in a clinical trial for patients with CRC. The Dynamic III trial will provide chemotherapy based on the presence or absence of ctDNA in post-operative CRC patients (ANZCTR, 2017).

While this review has focussed on ctDNA, there is the potential for other indicators of tumor presence to guide treatment decisions. These may include circulating tumor cells [but these are generally at very low levels in the blood (Saluja et al., 2018)], cell-free DNA, circulating tumor RNA, microRNA, nucleosomes and protein markers. Although CEA has low sensitivity for monitoring CRC, and its benefit to overall survival remains under debate (Primrose et al., 2014), other proteins show some promise, such as epidermal growth factor-like domain 7 (EGFL7) (Hansen et al., 2017). In addition, body fluids other than blood (e.g., urine, saliva, pleural fluid, cerebrospinal fluid, and stool) show potential for monitoring tumor biomarkers (Olmedillas-Lopez et al., 2017; Siravegna et al., 2017a).

## Conclusion

These studies highlight the potential utility of ctDNA in the ultrasensitive detection of residual CRC following a variety of treatment modalities, allowing for the selection of personalized and targeted therapy with optimal timing of delivery and early indication of the need to initiate or change therapeutic agents. This precision monitoring of patients prior to, during and following treatment shows enormous potential to transform the treatment of CRC. While many potentially useful ctDNA markers are available, more work is needed to determine which are best suited for specific purposes and for improving specific outcomes.

## Author Contributions

MR, HS, and ES planned the review topic, planned the inclusion and exclusion criteria, reviewed all articles and wrote and edited the manuscript. SV, CK, PH, and GY provided clinical advice on the content and reviewed and edited the manuscript.

## Funding

GY, CK, and ES are recipients of a grant funded by the financial support of Cancer Council SA’s Beat Cancer Project on behalf of its donors and the State Government of South Australia through the Department of Health together with the support of the Flinders Medical Centre Foundation, its donors and partners.

## Conflict of Interest

The authors declare that the research was conducted in the absence of any commercial or financial relationships that could be construed as a potential conflict of interest.
